# A mixed-signal implementation of a polychronous spiking neural network with delay adaptation

**DOI:** 10.3389/fnins.2014.00051

**Published:** 2014-03-18

**Authors:** Runchun M. Wang, Tara J. Hamilton, Jonathan C. Tapson, André van Schaik

**Affiliations:** Bioelectronics and Neuroscience, The MARCS Institute, University of Western SydneySydney, NSW, Australia

**Keywords:** mixed-signal implementation, polychronous spiking neural network, analog implementation, multiplexed neuron array, neuromorphic engineering

## Abstract

We present a mixed-signal implementation of a re-configurable polychronous spiking neural network capable of storing and recalling spatio-temporal patterns. The proposed neural network contains one neuron array and one axon array. Spike Timing Dependent Delay Plasticity is used to fine-tune delays and add dynamics to the network. In our mixed-signal implementation, the neurons and axons have been implemented as both analog and digital circuits. The system thus consists of one FPGA, containing the digital neuron array and the digital axon array, and one analog IC containing the analog neuron array and the analog axon array. The system can be easily configured to use different combinations of each. We present and discuss the experimental results of all combinations of the analog and digital axon arrays and the analog and digital neuron arrays. The test results show that the proposed neural network is capable of successfully recalling more than 85% of stored patterns using both analog and digital circuits.

## Introduction

Increasing evidence has been found that the mammalian neural system uses spatio-temporal coding in at least some of its operations (Van Rullen and Thorpe, 2001; Masuda and Aihara, 2003), largely due to this coding's potential to reduce energy consumption (Levy and Baxter, 1996). An artificial network that can learn and recall spatial and temporally encoded spike information will have significant benefits in terms of modeling these biological systems.

**Figure 1:**
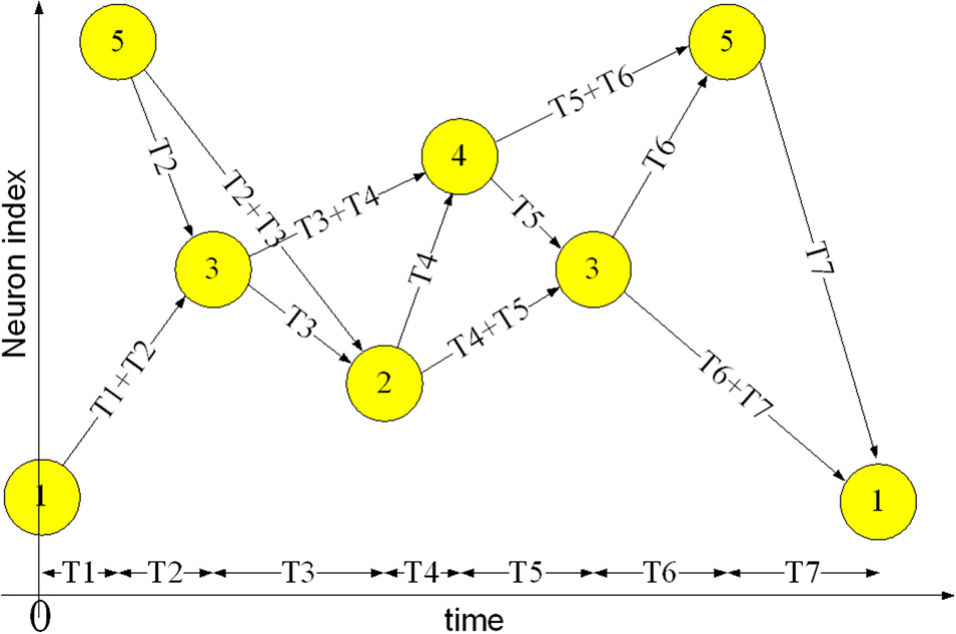
**Example of a spatio-temporal pattern**. The neurons fire asynchronously while their spikes arrive at the destination neurons synchronously, after traveling along axons with appropriate delays. This time-locked relation is the key feature of the spatio-temporal patterns.

A polychronous spiking neural network is a candidate for implementing a memory for spatio-temporal patterns. Polychronization is the process in which spikes travel down axons with specific delays to arrive at a common target neuron simultaneously and cause it to fire, despite the source neurons firing asynchronously (Izhikevich, 2006). This time-locked relation between the firing of different neurons is the key feature of spatio-temporal patterns. Neural networks based on this principle are referred to as “polychronous” neural networks and are capable of storing and recalling quite complicate spatio-temporal patterns. Figure 1 shows an example of a spatio-temporal pattern involving five neurons. The threshold voltage of each neuron is set so that it will fire if two pre-synaptic spikes arrive simultaneously. Whenever a neuron fires, its spike is transmitted to all connected neurons via its axonal connections, each of which has its own independent delay. These spikes will then generate post-synaptic currents at the connected neurons. The example pattern starts when neuron 1 fires at time 0 and neuron 5 fires at time T1. The spikes from both neurons will arrive at neuron 3 at time T1+T2, and together they will induce neuron 3 to fire at time T1+T2. In the same manner, the spikes from neuron 5 and neuron 3 arrive at neuron 2 simultaneously at time T1+T2+T3 and will cause neuron 2 to fire. This process will continue as long as at least two spikes arrive simultaneously at a neuron in the network.

Izhikevich (2006) calls these spatio-temporal patterns groups, and concludes that “spiking networks with delays have more groups than neurons” after presenting a network developed based on this polychronous principle. The groups in Izhikevich's network emerge in a randomly connected network of spiking neurons with axonal delays, following persistent stimulation and Spike Timing Dependent Plasticity (STDP) (Gerstner et al., 1996). However, one of the open problems of the theoretical model is to find patterns (groups): “Our algorithm for finding polychronous groups considers various triplets firing with various spiking patterns and determines the groups that are initiated by the patterns. Because of the combinatorial explosion, it is extremely inefficient” (Izhikevich, 2006). The method used by Izhikevich will take months of simulation time just to find these spatio-temporal patterns. Moreover, the polychronous groups emerge randomly and the same stimulus is not likely to result in the same polychronous groups every time. This makes the Izhikevich polychronous network unsuitable for practical applications such as pattern recognition. Finally this model is not efficient for hardware implementations, which we will discuss in detail in section Discussion.

To solve the problems presented above, we have proposed a digital implementation of a reconfigurable polychronous spiking neural network that can, in real time, learn specific patterns, and retrieve them (Wang et al., 2013b). Furthermore, our proposed polychronous neural network can use all the available hardware resources to store patterns. Test results show that the proposed neural network is capable of successfully recalling more than 95% of all spikes for 96% of the stored patterns. Unlike biological neural networks, the digital implementation is totally free of mismatch and noise. Therefore, we also designed an analog implementation, which is naturally subject to process variation and device mismatch, and which more closely emulates the analog computation in biological neurons.

Mixed-signal implementations of spiking neural networks benefit from many of the advantages of both analog and digital implementations. Analog implementations can realize biological behaviors of neurons in a very efficient manner, whereas digital implementations can provide the re-configurability needed for rapid prototyping of spiking neural networks. As a result, mixed-signal implementations offer an attractive neural network and many designs have been proposed for such systems (Goldberg et al., 2001; Gao and Hammerstrom, 2007; Mirhassani et al., 2007; Vogelstein et al., 2007; Harkin et al., 2008, 2009; Schemmel et al., 2008; Saighi et al., 2010; Yu and Cauwenberghs, 2010; Zaveri and Hammerstrom, 2011; Minkovich et al., 2012).

These proposed systems tend to employ programmable devices such as FPGAs and ASICs to route the spikes between analog computation modules. Some programmable platforms using floating gates (Basu et al., 2010; Brink et al., 2013). Furthermore, most of these systems use DACs to configure the analog modules to emulate different biological behaviors. Implementations of spiking neural networks with time-multiplexed analog circuits are described in Mirhassani et al. (2007), Yu and Cauwenberghs (2010), Minkovich et al. (2012) and a version that uses nanotechnology is described in Gao and Hammerstrom (2007), Zaveri and Hammerstrom (2011).

Here, we report on a mixed-signal platform, which combines both our analog and digital implementations and provides test results. Section Proposed Polychronous Network gives an overview of the proposed polychronous neural network. Section Design Choice presents the design choices that have been made for the neuromorphic implementation of the proposed polychronous network. The analog building blocks of the polychronous network (i.e., the neurons, axons, and other analog components) are detailed in section Analogue Implementation. Section Mixed-signal Implementation presents the proposed mixed-signal implementation, which includes the multiplexed analog neuron array and the interface between the asynchronous communication of the analog array and the (synchronous) FPGA. Measured results and a comparison to the fully digital implementation are given in section Results. In Section Discussion we discuss the performance of the different implementations and the key elements that influence the capacity and scaling of electronic realizations of polychronous networks and we conclude in section Conclusions.

## Materials and methods

### Proposed polychronous network

#### Training and recalling patterns

Two procedures are needed to use our proposed polychronous network to memorize and recall spatio-temporal patterns. The first is a training procedure in which the connection delay values of the axon paths between neurons are configured in order to meet the required timing relations of a given pattern. The second is a recall procedure, needed to retrieve a pattern that has been stored in the neural network through training. A pattern can be recalled by presenting the first few spikes of the pattern to the network, after which the network will complete the pattern if it is recognized. For example, to recall the example pattern shown above, neuron 1 needs to fire at time 0 and neuron 5 needs to fire at time T1. Together they will cause neuron 3 to fire and the remainder of the pattern will be induced by the network. The network is also capable of recalling parts of patterns that start somewhere in the middle, e.g., neuron 2 firing at time T1+T2+T3 and neuron 4 firing at time T1+T2+T3+T4 will retrieve the remainder of the example pattern.

The goal of the training procedure is to assign appropriate connection delays to axons in the polychronous neural network so that it is able to recall a specific pattern. We propose two mechanisms, which are *delay programming* and *delay adaptation*, to implement this function. Delay programming relies on a connection storing the delay value between a spike from its input neuron and a spike from its output neuron when both are induced to fire by some external training signal. It is not a biologically plausible method, but it is efficient in training and reduces testing time in scenarios where the result will not be affected by the training method. We therefore commonly use it to initialize a network.

Inspired by STDP, we developed a *delay adaptation* method, Spike Timing Dependent Delay Plasticity (STDDP), to fine-tune the delays during the training phase. We decrease the delay value of one axon by a small amount if the destination neuron fires (generating the post-synaptic spike) before the pre-synaptic spike arrives (at the synapse of the destination neuron), and we increase the delay in the opposite case. This procedure is repeated until the pre-synaptic spike arrives at the synapse simultaneously with the post-synaptic spike being generated. In the training phase, delay adaptation causes the connections to attain the desired delays through repeated presentation of the desired spatio-temporal patterns. The *delay programming* method can be regarded as a special case of the *delay adaptation* method in which the delay adaption is completed in just a single step and the delay is never altered subsequently. With the *delay adaptation* method, every time a pattern is recalled the delay values in the pattern will be updated, allowing the learned delays to be modified over time. Hardware implementations of non-polychronous networks that also adapt axonal delays can be found in (Hussain et al., 2012, in press).

#### Neural network structure

**Figure 2:**
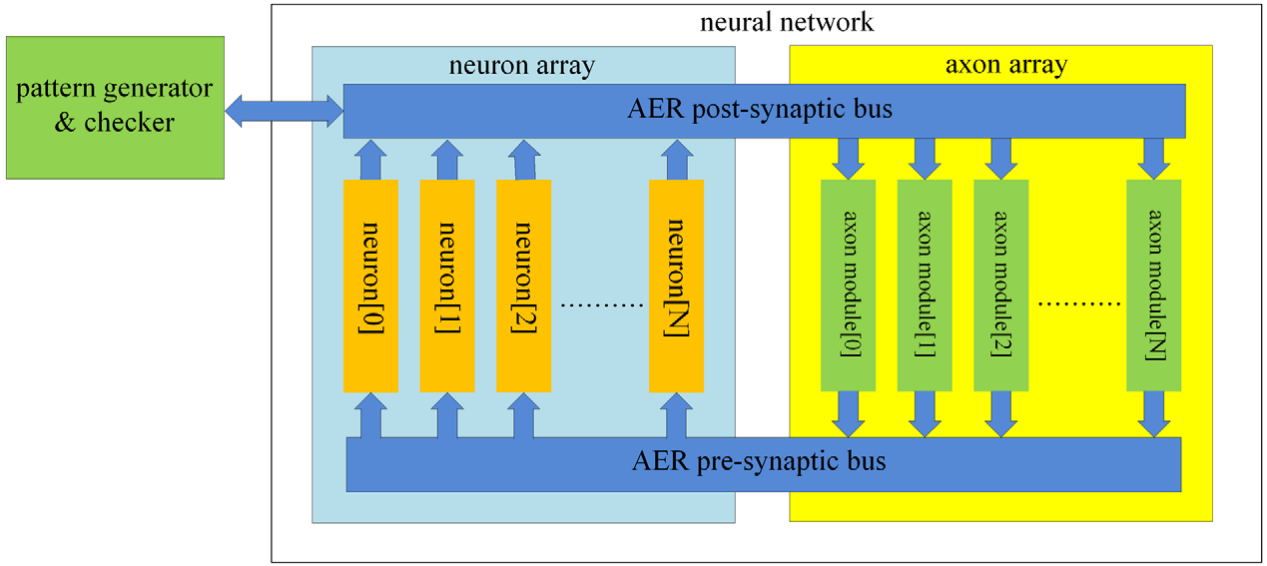
**Structure of the proposed polychronous neural network**. The neuron array generates post-synaptic spikes and then sends them to the axon array, which propagates these post-synaptic spikes, with programmable axonal delays, and generates the pre-synaptic spikes at the end of the axons. These pre-synaptic spikes are sent to the neuron array to cause the neurons to fire. The connectivity and delay of all the axons in the axon array are configurable.

The structure of the proposed neural network is shown in Figure 2. It contains two functional parts: a “neuron array” and an “axon array.” The neurons and the axons communicate with each other via Address-Event Representation (AER) buses (Boahen, 2000). Each neuron in the neuron array is identical in structure and has a unique AER address. The axon modules in the axon array are also identical in structure, and have both a unique physical address (their position in the array) and configurable input and output addresses, to place an axon between two neurons. The axon modules generate pre-synaptic spikes, which are received by the neurons. The neurons will then generate post-synaptic spikes if more than a certain number of pre-synaptic spikes arrive simultaneously. To decrease the likelihood of cross-talk between patterns, i.e., that a coincidence detecting neuron would be set off by a random coincidence, we used coincidence detectors with four inputs and a threshold of three spikes (Wang et al., 2013b).

The post-synaptic spikes are sent to the axon modules in the axon array. The axon array propagates these post-synaptic spikes with axonal-specific delay values and generates pre-synaptic spikes at the end of the axons. In the proposed neural network, the communication between any two neurons must be conducted via the axon modules in order to implement the polychronous network. This axon array, with reconfigurable input and output addresses, is capable of achieving much higher resource utilization than the method we have used previously (Wang et al., 2011), which generated spatio-temporal patterns based on fixed connectivity between neurons. That approach always resulted in networks where some axons remained unused. Our current approach is to generate delay paths *de novo*, so that only connections that actually appear in the training patterns will be created, by configuring the appropriate input and output addresses for each axon. Additionally we configured the system such that there can be any number of axonal delay paths between any two neurons in the network. In other words, several axons can have identical input and output addresses, placing them between the same two neurons. They would still be able to have different delay values, so that a spike originating from the input neuron would arrive at the output neuron multiple times after different delays, emulating the case where a neuron makes multiple synapses with another neuron.

**Figure 3:**
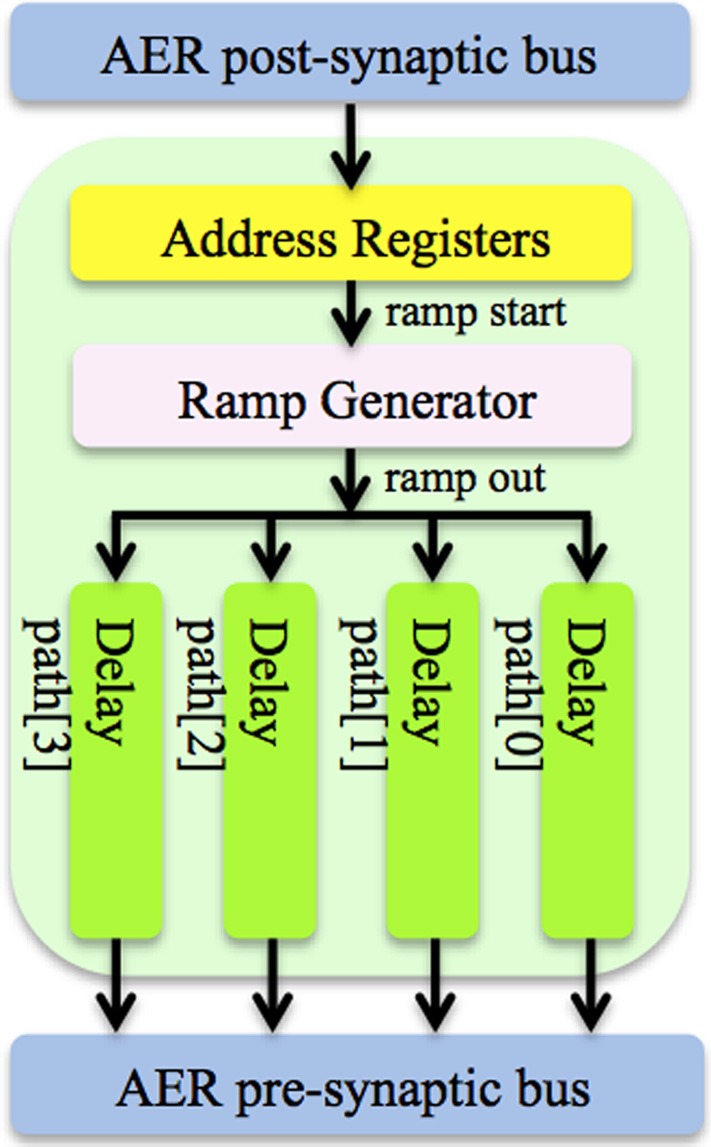
**Structure of the axon module**. The axon module receives post-synaptic spikes generated by the neuron in the neuron array via the AER post-synaptic bus. The axon module propagates these spikes with axonal-specific programmable delays and generates pre-synaptic spikes at the end of the axons. The address registers are used to store the input address and the four output addresses for the axonal delay paths.

The axon module (see Figure 3) has five address registers, one ramp generator, and four identical axonal delay paths. The address registers are used to store the input address and the four output addresses for the axonal delay paths. To place one axon module between neurons, we need to configure its address registers. At the beginning of the training, axon module[0] (see Figure 2) is enabled and all the other axon modules are disabled. When the first post-synaptic spike in a training pattern arrives, axon module[0] will latch the address of this spike as its input address and enable axon module[1]. The output addresses will be configured after the input address is configured. As there are four output addresses, one for each of the destination neurons, it will take four iterations for one axon module to finish the configuration of its output addresses (using the addresses of the next four sequential post-synaptic spikes in the training pattern after its input address is configured).

Delay programming is carried out in the same way as the address configuration. When the first post-synaptic spike arrives at axon module[0], it will start a ramp generator, which will send its value (ramp_out) to the four axonal delay paths. The delay of each axonal delay path is programmed when the output addresses are being configured (i.e., when the next four sequential post-synaptic spikes from the training pattern arrive). After delay programming, when a post-synaptic spike arrives and its address matches the input address of one axon module, it will start the ramp generator again. The axonal delay path will compare the output of the ramp generator with the programmed delay. A pre-synaptic spike will be generated when the output of the ramp generator exceeds the programmed delay with an address as stored in the output address register. The delays can also be configured using delay adaptation rather than delay programming. In this case the axonal delay is increased or decreased based on the delay between pre-synaptic spike and post-synaptic spike by using one of the three strategies: exact correction of the delay error in one step, correction of the error by a fixed amount each time, or correction by an amount proportional to the error. We have implemented all three strategies in the digital axon module. The first method is identical to just using the delay programming method. The second method, which uses a small fixed step, is very slow and produces similar results to the third method with a coefficient of 0.5. The digital axon presented here uses the third strategy. Slightly differently, the delay of the analog axon is programmed in an initial phase followed by a number of iterations of delay adaptation with a fixed update step, which was the simplest method to implement. An analog implementation that implements all three strategies would be too large for practical implementation on silicon.

### Design choice

#### Topology

**Figure 4:**
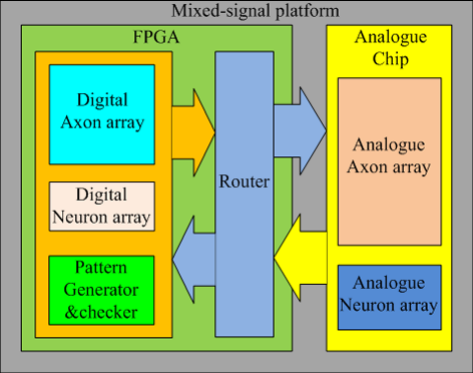
**Topology of the mixed-signal platform**. The FPGA contains the digital axon and neuron array, a router to control the destinations of spikes on the bus, and a pattern generator and checker for testing purposes. A separate IC contains the analog implementations of the axon and neuron arrays.

Figure 4 shows the topology of the proposed mixed-signal platform. It consists of one FPGA and one analog chip containing an analog neuron array and an analog axon array. The FPGA contains the digital axon array, the digital neuron array, a pattern generator and checker module for training and testing, and a router. The function of the router is to remap the addresses of the spikes between the digital implementation and the analog implementation; but in practice the router also needs to synchronize the spikes from the analog circuits before it can remap the addresses for these spikes. This is due to the analog circuits operating asynchronously and therefore without a clock, whereas the router is a fully digital design, which does require a clock. The spikes from the analog circuit therefore have to be synchronized to the clock domain in which the router works. We will present the design of an interface circuit for synchronization, followed by a circuit to implement the address remapping in section Synchronization Interface Circuit.

The system contains two types of implementations for the axon array and two for the neuron array, resulting in four potential combinations, which are presented below:

1. *A digital axon array and a digital neuron array*: This is simply the default FPGA implementation.
2. *Digital axon array and analog neuron array*: In this configuration, the router is required to re-map the addresses of the spikes transmitted between the analog neuron array and the digital axon array.
3. *Analog axon array and digital neuron array:* In this configuration, the router is also required to re-map the addresses of the spikes transmitted between the digital neuron array and the analog neuron array.
4. *Analog axon array and analog neuron array*: Despite having only analog implementations, the router is still required to transmit spikes between the analog axon array and the analog neuron array, as the addresses still require remapping. This is done to multiplex the analog neurons, so that inactive neurons in the network are not using hardware resources. This increases the size of the analog neuron array significantly. We will present the details of this approach in section Mixed-signal Implementation.

The neurons in the neuron array work as coincidence detectors that detect how many pre-synaptic spikes have arrived simultaneously. The FPGA implementation of these neurons uses four timers and one comparator (see Wang et al., 2013b). The analog version of these neurons is implemented using simple Leaky Integrate and Fire (LIF) neurons, which will be described in detail in section Analog Neuron Array. Since no complicated biological behaviors, such as spike rate adaptation or bursting, are required for the neurons in a polychronous network, we chose to implement LIF neurons, instead of more complex neuron models, e.g., the Izhikevich neuron model (Izhikevich, 2003) and the Mihalas-Niebur neuron model (Mihalaş and Niebur, 2009), to keep the size of the neuron circuit to a minimum.

For the axon module, the FPGA implementation uses a counter to implement the ramp generator, and registers to store the delay values. In the analog implementation, the ramp generator is implemented with a circuit that starts charging a MOS capacitor after receiving a spike on the AER bus. The axonal delay is generated by comparing a programmable voltage, stored on a capacitor, with the output signal of the ramp generator. The design and implementation of the ramp generator and the delay path can be found in (Wang et al., 2013a).

#### AER bus

There are two different AER buses in the proposed neural network: the AER post-synaptic bus and the AER pre-synaptic bus. The first is used to transmit post-synaptic spikes generated by the neurons to the axon modules. The second is used to transmit pre-synaptic spikes generated by the axon modules to the neurons (see Figure 3). The AER bus and protocol used in this system differs slightly from the standard AER bus and protocol (Boahen, 2000). We do not use handshaking, so we have omitted the request and acknowledge signals. Instead we use “active” lines to tell the receiver (neurons or axon modules) that a spike has been placed on the bus. Each neuron receives input from four neurons via four axons in our network. The pre-synaptic bus therefore uses four active lines, one for each synapse of the neuron. A further difference in our AER implementation is that there is no arbiter to deal with collisions when two addresses are placed on the bus simultaneously. We will address this issue in detail in section Discussion.

In our digital implementation, a single minimum-width binary address is used to reduce hardware costs, as the wiring for the bus will entail more resources than the implementation of the encoders/decoders in large scale FPGA designs (Harkin et al., 2008). This structure, however, doesn't satisfy our analog implementation, in which a full encoder/decoder will cost more area than the analog neuron itself in a 0.6 μm technology (typically each bit needs one XOR gate with 16 transistors in a full decoder). The AER buses in the analog neuron array use active lines and a 3/8-bit (three out of eight) address for which the encoding and decoding can be efficiently implemented in aVLSI, as will be shown in section Analog Neuron Array. The number of different addresses, *C*, for this code are given by the binomial coefficient:

(1) $$C_{M}^{N}=\frac{M!}{N!(M-N)!}$$

where *M* is the width of the bus and *N* is the number of bits that are HIGH in each address. In our implementation, *M* and *N* are set to 8 and 3, respectively, so that 56 addresses exist, which suffices for the size of our implementation. Both pre- and post-synaptic buses use this 3/8 bit code. The post-synaptic bus uses one active line in addition to the address to indicate an address has been placed on the bus, while the pre-synaptic bus uses four active lines—one for each of the four synapses an axon can target.

The addresses of the AER buses in the analog axon array are encoded in a format of 4 out of 9 high bits, yielding 126 addresses—one for each neuron. Increasing the bus width would allow more neurons at the cost of additional area for the bus and the decoder. The choice of 4/9 for this bus is a trade-off between performance and the cost of silicon.

### Analog implementation

#### Analog neuron array

**Figure 5:**
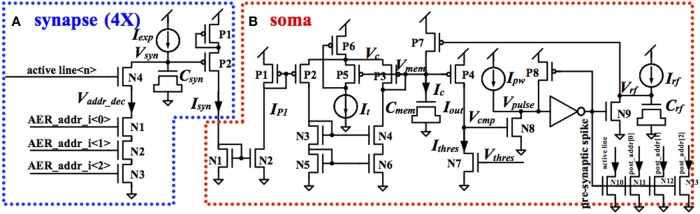
**Circuit diagram of the analog synapse (A) and soma (B)**.

The proposed LIF neuron comprises four identical charge-and-discharge synapses, one for each active line on the pre-synaptic bus. The structure of the synapse was first proposed by Arthur and Boahen (2004). Figure 5A shows the schematic of the charge-and-discharge synapse, which will generate a post-synaptic current for every incoming pre-synaptic spike. This synapse comprises a reset circuit (N1-N4), a MOS capacitor (*C_syn_*, ~100 fF), a voltage-to-current conversion circuit (P1-P2) and a DC current source (*I_exp_*, set to 12 pA).

The 3/8 high bits of the pre-synaptic address are connected to N1-N3. On arrival of a pre-synaptic spike with these three bits HIGH and the appropriate active line high, N1-N4 will conduct and pull *V_syn_* down to ground. After that, *V_syn_* will be pulled up to *V_dd_* by *I_exp_*. The voltage-to-current conversion circuit will transduce *V_syn_* into *I_syn_*, the post-synaptic current, which will decay exponentially, due to the linearly increasing *V_syn_*. To reduce power consumption, P1, a diode connected pMOS transistor, is added to limit the gate-source voltage of P2. *I_syn_* will be injected into the soma for integration. All four synapses of a LIF neuron are identical, using the same 3/8 bit address, but are connected to different active lines.

Figure 5B shows the schematic of the soma. The post-synaptic currents from four synapses are sent to a current mirror (N1-N2) for summing. The current mirror will convey *I_syn_*, the sum of the post-synaptic currents, to *I*_*P*1_, which is the input current of a first-order low-pass filter. Furthermore, by changing the width/length ratio of N1 or N2, the input current to the low pass filter can be easily scaled or amplified.

The low-pass filter, which was first proposed in Python and Enz (2001), is the basic building block of the soma. In our previous work (Wang et al., 2011), we have shown that its output current *I_out_* has the following equation:

(2) $$\tau_{mem}\frac{dI_{out}}{dt}+I_{out}=I_{P1}$$

where the time constant of the implementation is given by:

(3) $$\tau_{mem}=\frac{nU_{T}C_{mem}}{I_{t}}$$

where *U_T_* is the thermal voltage, *n* is the weak inversion slope factor, and *I_t_* is a DC current source (set to 1 nA). More details can be found in Wang et al. (2011).

To generate the post-synaptic spike, the output current of this low-pass filter *I_out_* is compared with a constant current *I_thres_* introduced by N7. The value of *I_thres_* is set by *V_thres_* to a value such that three pre-synaptic spikes arriving within 1 ms will make *I_out_* strong enough to pull *V_cmp_* up to *V_dd_*. When *V_cmp_* exceeds the threshold of N8, N8 will conduct and pull *V_pulse_* down to ground. *V_pulse_* is sent to an inverter to generate the post-synaptic spike. It is HIGH when *V_pulse_* is lower than the threshold of the inverter.

The refractory period is implemented by a circuit composed of N9, P7, a MOS capacitor (*C_rf_*, ~100 fF) and a DC current source (*I_rf_*, set to 12 pA). When the post-synaptic spike is HIGH, N9 will conduct and pull *V_rf_* down to ground. After that, *V_rf_* will be pulled up to *V_dd_* by *I_rf_*. P7 will conduct and pull *V_mem_* up to *V_dd_* when *V_rf_* is lower than the threshold of P7. The time when *V_mem_* is at *V_dd_* is the refractory period, during which the low-pass filter will not do any integration. Since this refractory time is active when *V_rf_* is lower than the threshold of P7, the refractory time is thus controlled by the size of *C_rf_*, the capacitor, and *I_rf_*, the charging current.

When *V_mem_* is pulled up to *V_dd_* and *I_out_* is reset to 0, *V_cmp_* will be pulled down to ground by *I_thres_*. N8 will stop conducting when *V_cmp_* is low and *V_pulse_* will then be pulled up to *V_dd_* by a constant current *I_pw_*. The post-synaptic spike, which is the inverted signal of *V_pulse_*, will then be reset. A feedback circuit (P8) will pull *V_pulse_* up to *V_dd_* quickly once *V_pulse_* exceeds the threshold voltage of the inverter, to reduce power consumption. The pulse width of the post-synaptic spike, which is the time when *V_pulse_* is lower than the threshold of the inverter, is controlled by *I_pw_*, which is used to pull *V_pulse_* up.

An address encoder (N10-N13, using four minimum-sized nMOS transistors to drive the active line and 3/8-bit address of the AER post-synaptic bus), will convert the voltage-mode post-synaptic spike into a current-mode spike. The current-mode spike will be sent to the AER post-synaptic bus. As the AER post-synaptic bus needs to be driven in parallel by all the analog LIF neurons, an implementation with voltage-mode spikes would need a high fan-in OR gate or an arbiter, which would take up a significant amount of area in the layout. Furthermore, using voltage-mode spikes for on-chip routing will take up significant area as each spike needs one wire, whereas the current-mode spikes can share one bus, e.g., one wire can be shared by the active lines from all the 50 neurons.

As a trade-off between fabrication cost and the size of the neuron array, we chose to implement 50 analog LIF neurons in the analog neuron array, which led to the choice of the 3/8-bit address format. The layout of the analog LIF neuron is as compact as possible and all signals are routed across the neuron. In this way, the placement of the neurons in an array is quite straightforward; the neurons are placed in one row.

All transistors are 2.4 μm wide and 3.6 μm long (P8, N3, N4, and N8 is 0.6 μm long, N1 is 4.5 μm wide and P7 is 4.8 μm wide and 0.6 μm long). The inverter I1 use transistors are 2.4 μm wide and 0.6 μm long. The MOS capacitor values are: *C_mem_* = 15 × 24 μm (~0.6 pF) and *C_rfc_* = 3.6 × 2.4 μm (~0.02 pF). In the layout of the neuron array, for each neuron, we just need to connect the three transistors that form the address decoder (N1-N3) in the current synapse (see Figure 5A), to three bits in the address of the AER pre-synaptic bus according to the unique 3/8-bit address of that neuron. An active line on the AER pre-synaptic bus is connected to N4 of a current synapse. Each of the four current synapses will have its own active line on the AER pre-synaptic bus. Similarly, for each neuron, we just need to connect the four transistors, which compose the address encoder (N10-N13) in Figure 5B, to the active line and to the three high bits in the address on the current-mode AER post-synaptic bus according to the unique 3/8-bit address of that neuron. In this way, the layout of the neuron array will remain compact as no extra routing of the AER buses is needed.

#### Analog axon array

The structure of the analog axon module is shown in Figure 3. It comprises three parts: a ramp generator, four axonal delay paths and an AER interface circuit. The AER interface circuit carries out the function of the address configuration, the address decoding and the address encoding. The ramp generator will start when receiving a spike on the AER bus. The details of the design and implementation of the ramp generator and the delay path can be found in Wang et al. (2013a).

**Figure 6:**
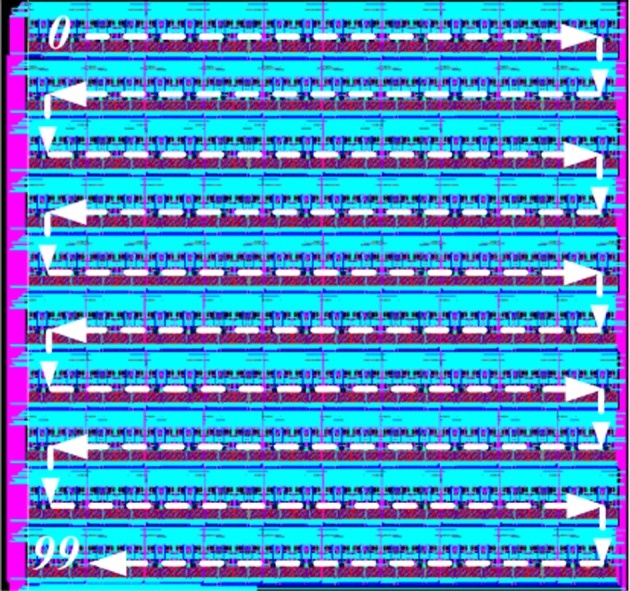
**Layout of the axon array**. Arrows show how the axons modules are placed in a 1-D array.

The analog axon array contains 100 identical analog axon modules connected serially. Due to the size of the axon module, we cannot place these 100 axon modules physically in one row (it would be 20 mm long) but instead the array is folded to create a 10×10 2-D array, as shown in Figure 6. As in the layout of the neuron module all the AER buses, control signals, and bias currents are routed horizontally across the axon module so that neighboring neurons in a row are simply connected by placing them next to each other. The horizontal buses in each row are connected to two vertical buses placed on both sides of the axon array for interconnection. As for the neuron array, the spikes generated by the axon modules are all current-mode spikes within the chip and they are converted to voltage-mode spikes for off-chip transmission.

### Mixed-signal implementation

#### Multiplexed analog neuron array

The motivation for developing a multiplexed analog neuron array is to increase the size of the analog neuron array without increasing the cost of the system significantly. A polychronous neural network composed of a neuron array with 50 neurons will suffer from severe cross-talk between patterns, which occurs when a neuron belonging to one pattern fires accidently as a result of pre-synaptic spikes from other patterns or another part of the same pattern. The effect of cross-talk depends on the overlap (correlation) of the patterns and can be regarded as noise. The more overlap there is, the higher the possibility that a pattern plus some noise spikes will also set off a different pattern. Also, the more input connections a neuron has, i.e., the more patterns this neuron is a member of, the more likely this neuron is to get three simultaneous inputs as a result of noise. In severe cases of cross-talk, all neurons in the network will fire continuously in an uncontrolled manner. To mitigate this problem, we need to increase the sparsity of the neural network, i.e., decrease the number of patterns to which each neuron is sensitive. This can be achieved by increasing the size of the neuron array, as the patterns generated by the pattern generator are evenly distributed over the whole network. The conventional approach to increase the size of the analog neuron array is to simply add more physical neurons. As expected, hardware costs increase linearly in relation to the size of the neuron array if all the neurons are to be implemented physically.

Inspired by the multiplexed neuron array used in the digital implementation (Wang et al., 2013b), we propose a similar approach to implement a multiplexed analog neuron array. We can use the fact that in a typical polychronous network, only a small percentage (less than 5%) of the neurons are active at any given time, and only those active neurons need to be physically implemented.

**Figure 7:**
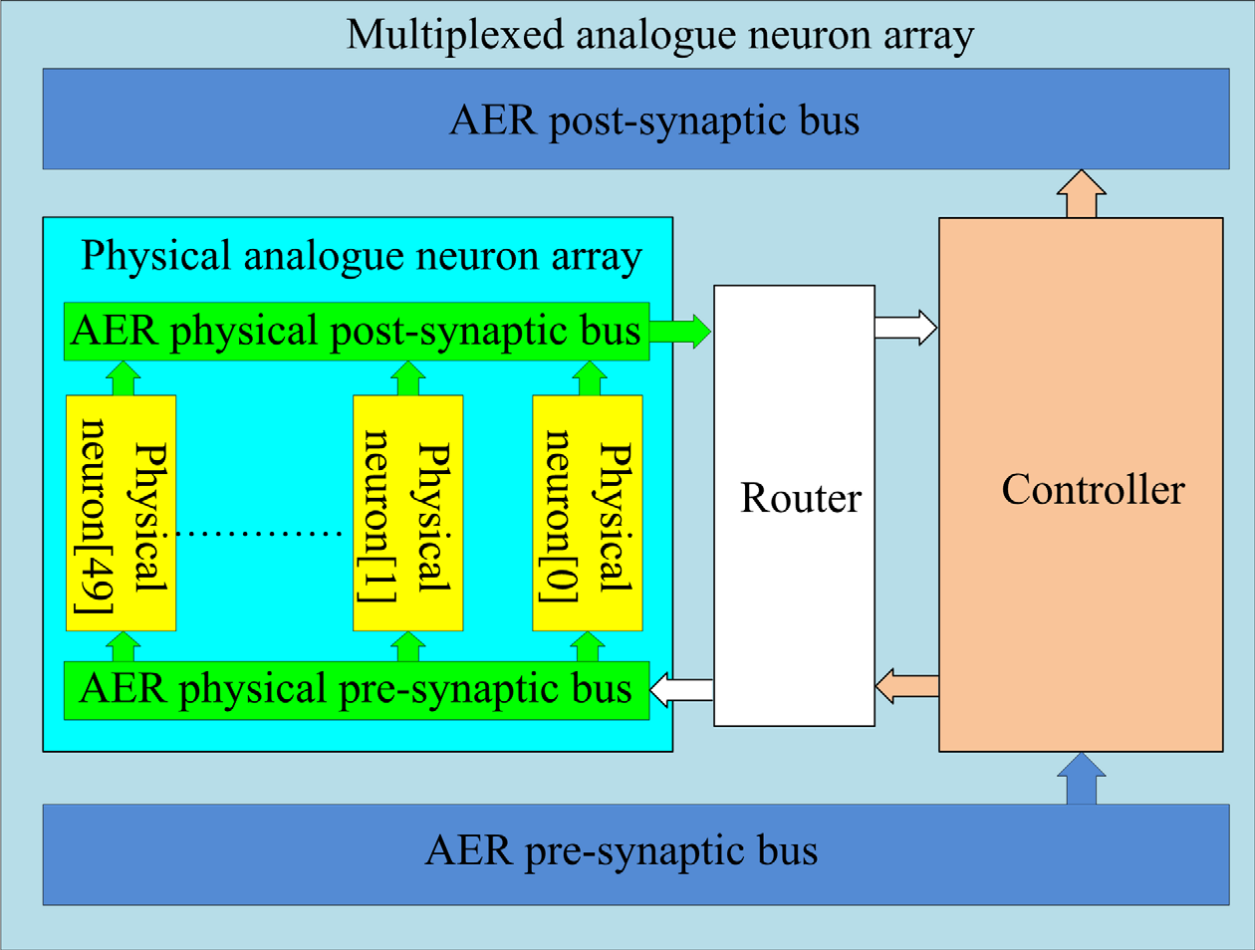
**Structure of the multiplexed analog neuron array**. The controller and router map virtual addresses from the AER busses to physical addresses on the analog neuron array, so that only active neurons in the network are using hardware resources.

The structure of the multiplexed analog neuron array is shown in Figure 7. It consists of two sub-blocks: a physical neuron array and a controller. They communicate with each other via two internal AER buses: the AER physical pre-synaptic bus and the AER physical post-synaptic bus. The controller receives pre-synaptic spikes from the axon array and assigns them to the physical neurons for the generation of post-synaptic spikes, which will be sent to the axon array. From the point of view of the axon array, the multiplexed neuron array appears as a neuron array with 4k neurons. The addresses of the spikes between the controller (a single minimum-width binary address) and the analog neuron array (the 3/8-bit address format) need to be remapped by the router, which will also synchronize the spikes from the analog circuits. For simplicity, in the following description, we assume the controller is connected to the analog neuron array without synchronization and address remapping.

The controller dynamically assigns analog neurons to each incoming pre-synaptic spike. The analog neurons are used to detect how many pre-synaptic spikes have arrived within 1 ms of each other. When a spike arrives from the axon array and an analog neuron has already been assigned for that spike's address, the spike will be sent to that neuron. The address of this incoming spike will have been latched in a register linked to that analog neuron. If no neuron has been assigned for the arriving address, the spike will be sent to an unassigned neuron, which will then be labeled as assigned by the controller, by latching the address of the spike. The controller will also start a timer linked to that analog neuron. Once the timer of that neuron has expired (after 1 ms), the neuron will be freed and labeled as unassigned by the controller. When a post-synaptic spike is generated by an analog neuron, the controller will send it to the axon array with the address that is stored in its register. More details about the controller can be found in Wang et al. (2013b).

Based on this structure, a neuron array with 4k virtual analog neurons can be achieved using only 50 physical neurons. This multiplexed analog neuron array is thus 80 times more efficient in silicon area on the analog side. It does, however, require a controller implemented on an FPGA. This does not increase the cost of the system significantly as the FPGA is needed anyway to carry out other tasks such as pattern generation, address remapping and other miscellaneous tasks. Furthermore, this mixed-signal implementation offers a much higher degree of extensibility as the LIF neurons used in this implementation could easily be replaced with other neuron models if desired.

#### Synchronization interface circuit

To use the asynchronous analog circuits with the FPGA, synchronization with its clock domain is needed. In digital circuit design, a general method used to do this is to use two (or more) serially connected flip-flops to sample the input (Weste and Harris, 2005). This scheme works well for 1-bit signals but it does not extend to catering for parallel signals, such as the address bus and data bus, due to potential timing skew on these buses that could cause each bit in the bus to arrive at a slightly different time. This can lead to race conditions and hazard problems, and can ultimately lead to the wrong address being sampled (Weste and Harris, 2005).

In our design, this timing skew comes from two sources. The first is the analog circuit that converts the current-mode spikes to voltage-mode spikes. Due to process variation and parasitic capacitors between the wires and transistors, the conversion for each line of the bus will take a slightly different amount of time. For the very same reasons, the pulse width of each active line and each bit in the address will also be slightly different. The second source of timing skew is caused by the propagation delay of the signals along the tracks of the Printed Circuit Board on their way to the FPGA.

**Figure 8:**
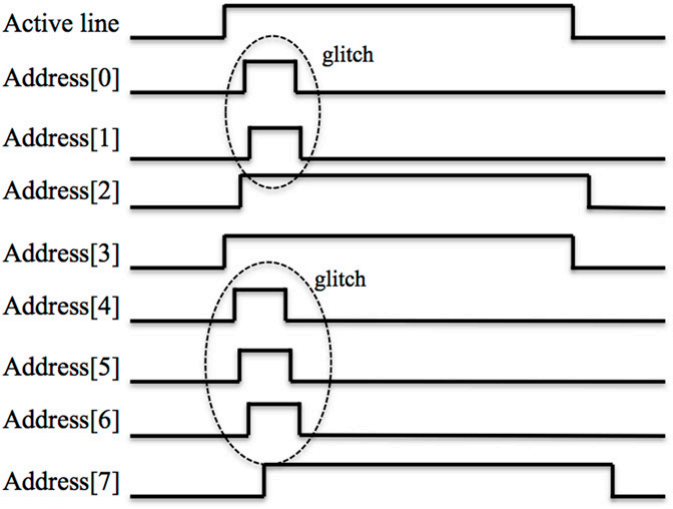
**Waveform of a spike from an analog neuron on the post-synaptic AER bus showing timing skew and glitches**.

Figure 8 illustrates a waveform of a post-synaptic spike from an analog LIF neuron (the waveform from the analog axon is quite similar). In the figure, the timing skew can clearly be seen as each bit in the bus arrives at a slightly different time. Besides the timing skew, there is also an additional problem in the form of glitches, which are brief digital pulses, up to tens of nanoseconds long. They are caused by the coupling capacitance between the wires and transistors. These glitches, in spite of their short period, are still likely to be sampled by the digital circuit (running at 50 MHz) and ultimately may lead to the wrong addresses being sampled.

One common method to minimize the timing skew caused by transistor mismatch is to use clocked flip-flops (Weste and Harris, 2005) to generate these spikes. We have not used this method because it would increase the design overhead of circuit and introduce another problem, namely that of synchronizing the clock signal of the chip and the FPGA. The timing skew caused by propagation delays on the PCB is usually minimized by carefully tuning the length of the tracks on the PCB. We have not used that method either as it would significantly increase the effort and cost of manufacturing the PCB.

In digital designs, the general way to sample an asynchronous parallel bus is to use a handshake protocol to guarantee that the receiver will only sample the data when the data is stable (Weste and Harris, 2005). In other words, the sender needs to inform the receiver when to sample the data. The drawback of this method is that it requires extra logic circuits on both the sender and the receiver. In cases where there is more than one sender on the bus trying to send data, some form of arbitration is required, further increasing the circuit complexity and the cost of hardware resources.

Instead of the above methods, we chose to synchronize the spikes from the analog implementations by using an interface circuit to carry out the synchronization in three steps without requiring a handshake protocol. For illustration, we will use the AER bus of the analog neuron array in the following explanation. The interface circuit handles the AER bus of the analog axon array in the same way.

The first step is to synchronize each active line and each bit of the address of the incoming spike in the conventional manner by using a circuit composed of a serial connected flip-flop for each of them (four in total). The output values of the flip-flops for the address and active lines are referred to as the synchronized address and the synchronized active line, respectively. The address of the post-synaptic spike is encoded in the 3/8-bit format, which means that any address that does not have exactly three out of eight bits active is invalid.

The second step is then to latch the synchronized address and active line only when a valid address is present, i.e., when exactly three bits are HIGH, and store it in a register. We have implemented this register as a 32×9 bit FIFO, using eight bits for the address and one bit for the active line. We use a counter to determine how many bits are HIGH in the synchronized address and we can distinguish two situations that need an action when a valid address is detected:

1. The arrival of a spike with a valid address when the address at the previous clock cycle was invalid. In this condition, the value of the counter in current clock cycle is three, whilst the value of the counter at previous clock cycle was not equal to three. The address of the spike is latched in the FIFO
2. The arrival of a spike with a valid address that is different from a valid address at the previous clock cycle. In this case, the value of the counter in the current clock cycle and previous clock cycle are both equal to three, whereas the value of the synchronized address in current clock cycle is not equal to the value at the previous clock cycle. The new address is stored in the FIFO.

In all other cases, including when a valid address is detected that is the same as in the previous clock cycle, the data on the bus is ignored. In this way, the asynchronous spikes from the analog neuron array are synchronized and stored in the FIFO. The third step is to generate spikes with a fixed pulse width (four clock cycles) by reading the FIFO. If the FIFO is empty, all the synchronized pre-synaptic spikes have been read out and no new spikes will be generated.

The interface circuit for the spikes from the analog axon array operates in the same way with the exception that a third condition needs to be handled:

3. The arrival of a spike with a valid address that is the same as the last one that arrived, but on a different synapse. In this case, the value of the counter in current clock cycle and previous clock cycle are both four (4/9-bit format) and the value of the synchronized address in both cycles is the same, but the value of the synchronized active lines is different. The new address and active line are stored in a 32×13 bit FIFO (nine bits for the address and four bits for the active lines).

The interface circuit effectively eliminate the problems of timing skew and glitches on the bus. It is also capable of sampling the asynchronous spikes from the analog circuits with a high temporal accuracy, as shown by the results that will be presented in section Performance of the Interface Circuit. For spikes that need to be sent to the analog chip, we use the conventional means of synchronizing them to the system clock by using flip-flops on the FPGA to minimize the timing skew on the address lines (Weste and Harris, 2005).

#### Address remapping

Address remapping is the second function of the router. The controller can be configured for multiplexed analog neuron arrays or multiplexed digital neuron arrays. When it is configured for a multiplexed analog neuron array, the router needs to carry out the remapping for the addresses of spikes traveling between the controller and the analog neuron array. To use the analog axon array, the router needs to carry out the address remapping for the spikes traveling between the analog axon array and the controller regardless of whether it is configured for multiplexed analog or digital neuron array.

The router was implemented using four look-up tables, one for each of the four address remapping possibilities. For spikes from the analog axon/neuron array, the router synchronizes them using the interface circuit first. These synchronized spikes are then compared to the look-up tables in order to convert their addresses to the corresponding binary-encoded addresses. These spikes are then sent to the controller for processing. Spikes generated by the controller are also compared against the look-up tables to convert their addresses to either 3/8-bit or 4/9-bit addresses. After being converted, these spikes are sent to the analog axon/neuron array.

## Results

The proposed polychronous neural network is designed to train and recall patterns rather than to randomly react to some spatio-temporal patterns (groups) that have emerged in the network, as is the case in Izhikevich (2006). Performance in our network is therefore measured as the rate of success in recalling the trained patterns. The advantage of our approach is that the network can be used as a memory that can learn spatio-temporal patterns. Furthermore this approach optimizes the use of the available hardware, so that in our approach all available neurons and axons in the hardware arrays can be used, while in the original polychronous network some neurons and many connections are not part of any pattern and thus never used. The disadvantage of our approach is that overlap between patterns (cross-talk) has to be limited and it is not possible to store near identical patterns.

There are four possible combinations of analog or digital axons and neurons. The fully digital (FPGA) combination implements the proposed neural network faithfully with hardly any effect of noise and process variations. The measurements form this combination therefore present the optimal performance of our polychronous neural network model. The results of all the other three combinations will be compared with the results of the fully digital implementation in the sections Digital Axon Array and Analog Neuron Array to Analog Axon Array and Analog Neuron Array. Section Performance of the Interface Circuit first discusses the performance of the interface circuit described in section Synchronization Interface Circuit.

### Performance of the interface circuit

Testing the interface circuit is the first step in testing the whole system. To obtain a direct measurement of the ability of the interface circuit to synchronize and latch addresses correctly, we use the FPGA to send a pre-synaptic spike to an analog neuron to induce it to fire. The interface circuit is then used to synchronize and latch the spike from the analog neuron with the FPGA's clock. We then compare the address of this latched post-synaptic spike with the expected address, as determined by which neuron the FPGA induced to fire. If their addresses match, this means the interface circuit works correctly.

Sometimes the interface circuit samples the same address twice. This is caused by glitches that can cause a valid address to become briefly invalid, when more than three address lines are high, before returning to the valid address as the glitches subside. This double sampling could be solved by adding an internal timer to the interface circuit to guarantee that an address could only be sampled once within a short period (say 1 μs). However, we have not employed this method as the second spike sampled will only cause a small offset (<1 μs) in the axonal delay, which starts on the arrival of a post-synaptic spike. This offset will not affect the performance of the proposed polychronous neural network at all.

**Figure 9:**
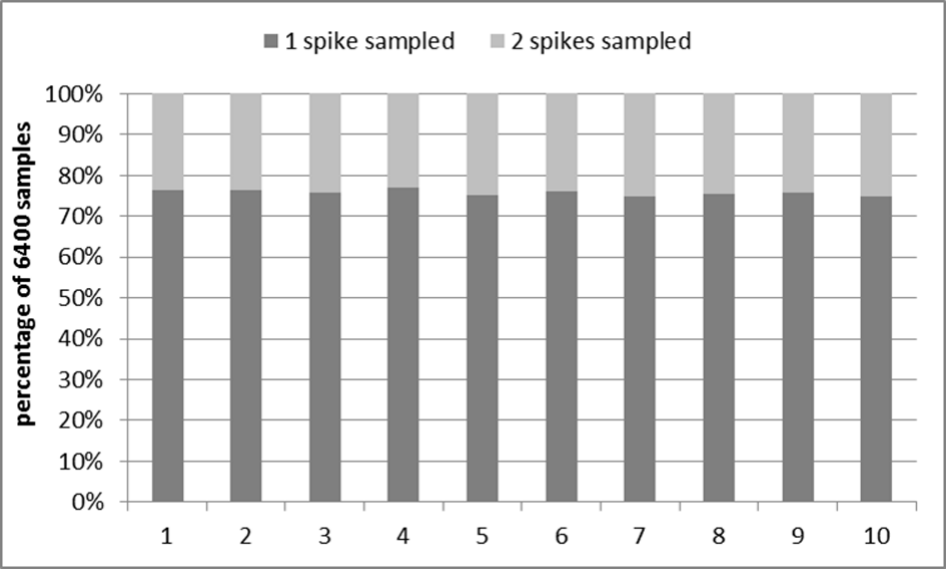
**Performance of the interface circuit**. Dark gray: valid address sampled once; Light gray: valid address sampled twice in succession.

Figure 9 shows the results of the tests. All 50 addresses (one for each analog neuron) were tested 128 times (with an interval time of 5 ms to guarantee there will be one post-synaptic spike each time). This test was then repeated 10 times. In each of the 10 runs, for approximately 75% of the time the correct address was sampled once while for the remainder of the cases, the correct address was sampled twice in succession. No wrong addresses were sampled in these tests.

### Digital axon array and analog neuron array

#### Delay programming

In the setup for the delay programming tests, a single axon array was used in the neural network, yielding 4k axon modules with 16k (16384) axonal delay paths (connections). Note that unlike in Izhikevich (2006), no connections are shared between two patterns, so that the number of available connections directly determines the maximum number of inter-spike intervals that can be programmed into our network. Each axon module contains four axonal delay paths (see Figure 3), and for each spike in the polychronous pattern, 4 delay paths are needed from the four previous spikes in the pattern. Thus, the number of the inter-spike intervals that our neural network can store is simply equal to the number of axon modules. If, for instance, the patterns to be stored each contain 50 inter-spike intervals, the maximum number of such patterns that can be stored in the neural network is 82 (4k/51).

**Figure 10:**
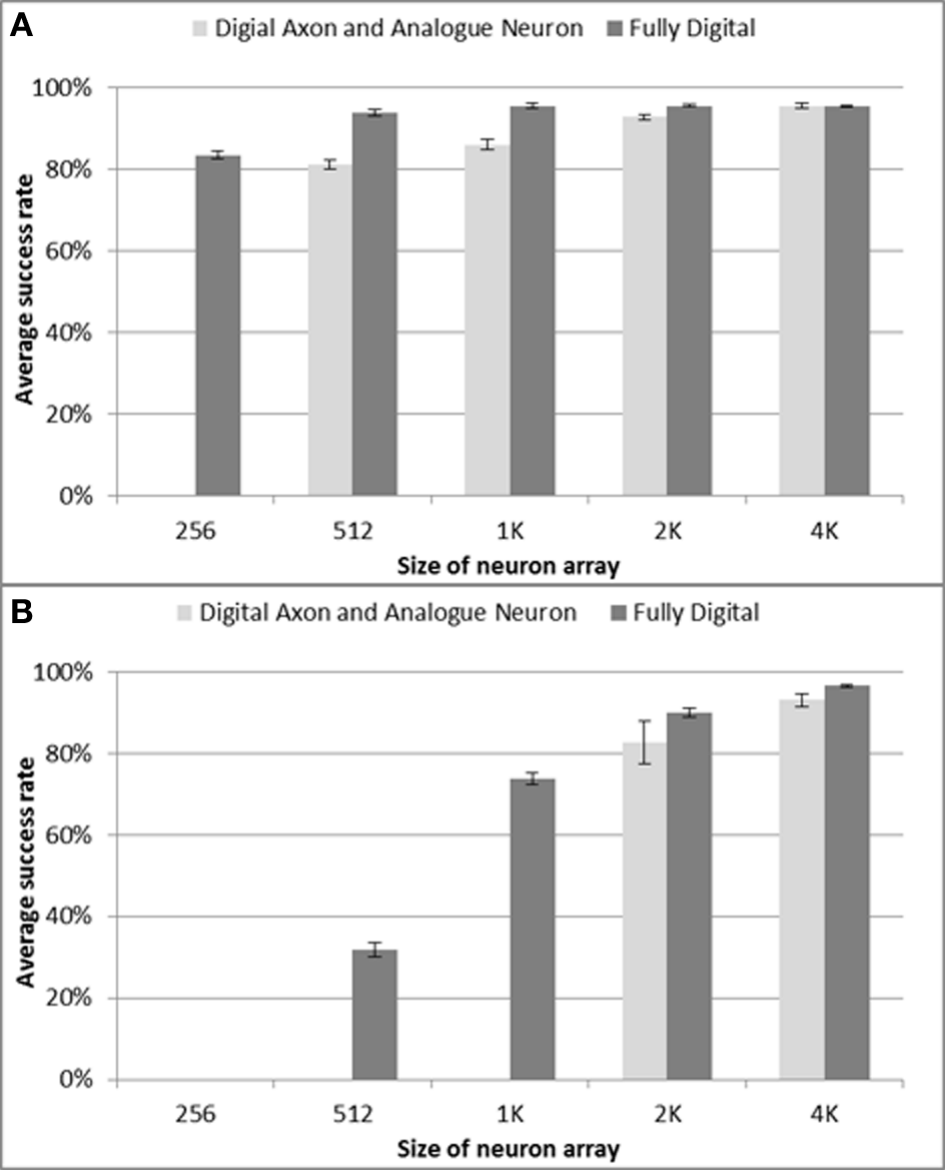
**Percentage of stored patterns successfully recalled for different neuron array sizes. (A)** delay programming and **(B)** delay adaptation, respectively. The results for the fully digital implementation are added for comparison purpose. Error bars are standard errors of the mean.

The patterns are trained only once when using delay programming. There is also only one recall test as there is no adaptation, and the result of a recall will be the same each time. For each configuration of the neural network, 10 test runs were conducted. The pattern generator & checker module generates spatio-temporal patterns for training and for testing whether the patterns can be recalled successfully. We tested neuron array sizes ranging from 128 to 4k neurons and test results are shown in Figure 10A. For the configurations consisting of 128 and 256 neurons (not shown in Figure 10A) and trained with 82 patterns having 51 spikes each, the neural network enters an all firing state in which all the neurons fire simultaneously, showing that a network of this size using analog neurons cannot cope with that number of patterns. In the digital implementation, this only happens for configurations consisting of 128 neurons, while a network with 256 neurons achieves an average success rate about 80%. To achieve a similar success rate when using analog LIF neurons, the network needs at least 512 neurons. Furthermore, the results for a network with 1k and 2k analog neurons are also slightly worse than their digital counterparts. Only the result for 4k analog neurons matches the digital implementation. As an aside, this proves that the proposed interface circuit is capable of sampling the asynchronous spikes from the analog circuits correctly, because otherwise the performance would be much worse than in the digital implementation.

The results indicate that the effects of cross-talk are more serious when using the multiplexed analog neuron array, so that a network with analog neurons performs worse than one with digital neurons when the size of the network is small. Due to process variation and device mismatch, the analog neurons cannot be perfectly tuned to all generate a post-synaptic spike only when at least 3 out of 4 pre-synaptic spikes arrive within 1 ms. In other words, the analog neuron is not as precise a coincidence detector as the digital neuron. Moreover, due to the parasitic capacitances on chip, the analog LIF neuron will sometimes generate spikes by accident, e.g., the firing of one neuron will trigger its neighboring neuron to fire, which increases cross-talk. Increasing the size of the network increases the sparsity (i.e., decreases the number of patterns to which a neuron belongs Wang et al., 2013b), and the difference in the performance between the analog neurons and the digital neurons will become negligible for larger networks.

#### Delay adaptation

In the tests for the delay-adaptation mode, each pattern was trained five times and recalled one time. The strategy used adapted the delay by half the time difference between the pre- and post-synaptic spikes each time a neuron fired. The same settings used in the delay programming scenario were used for these tests, but all delays were initialized with random values. We again tested neuron array sizes from 128 to 4k neurons and the test results are shown in Figure 10B. For the networks with a size smaller than 2k neurons, only a few patterns can be recalled successfully and their results are therefore not included in Figure 10B. The results in Figure 10 also show the performance drops more in delay adaptation mode than in the delay programming mode when compared with the digital implementation. This is again the result of the larger sensitivity to cross-talk in the analog neuron array.

#### Effect of noise

In this set of tests, random noise was injected into the network. The Poisson rate of the noise, generated by a LFSR, was varied from 2 to 128 spikes per second. This firing rate represents the number of additional spikes, i.e., not belonging to any of the trained patterns, presented to the network in a one second window. As each spike is generated by a randomly chosen neuron, the spike rate measures the total noise input, not the firing rate of individual neurons.

**Figure 11:**
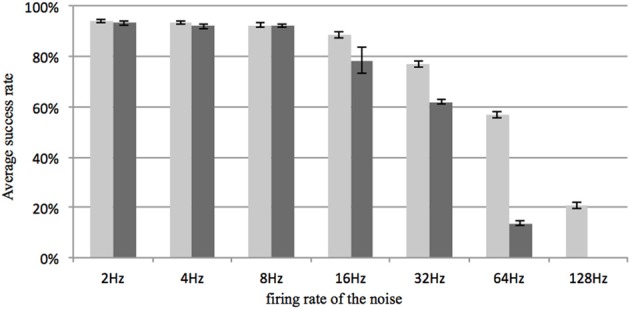
**Recall percentage for various Poisson rates of the noise generator**. The firing rate represents the total number of additional random spikes per second in the network. For comparison, the firing rate of a stored pattern is about 100 spikes per second (50 events in about 500 ms). Light gray: delay programming; Dark gray: delay adaptation. Error bars are standard errors of the mean.

All other settings were kept the same as in the delay-programming mode and the delay-adaptation mode with a neuron array consisting of 4k neurons. In both modes, no noise was added during the first training time. Figure 11 shows the result, which proves that the system is fairly robust to noise when the sparsity of the neural network is large.

#### Capacity for storing spatio-temporal patterns

**Figure 12:**
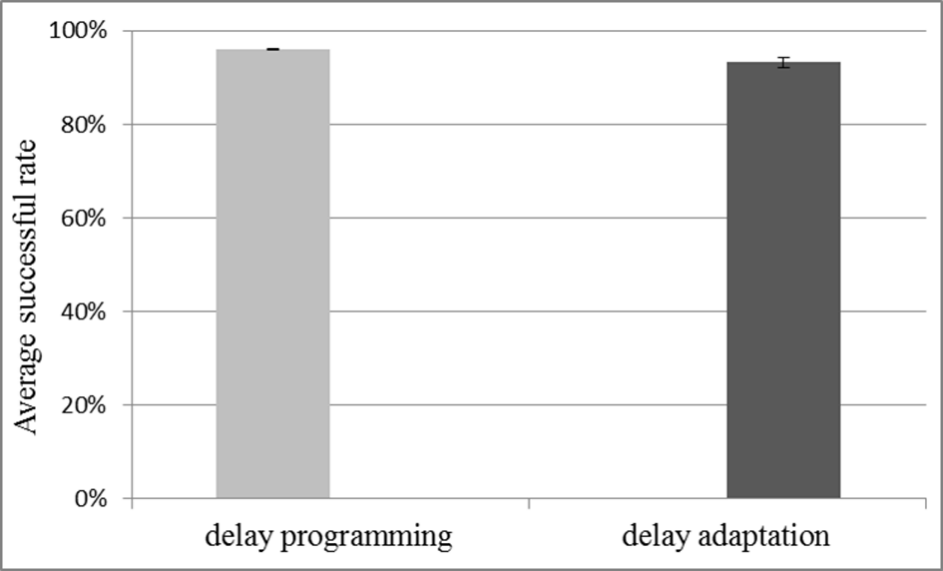
**Result for capacity testing with 1000 stored patterns of 51 spikes each**. The network consists of 4k neurons and 80k axon modules. Both methods of delay configuration resulted in approximately 95% of the stored patterns being successfully recalled. Error bars are standard errors of the mean.

To test the capacity for storing spatio-temporal patterns when using the multiplexed analog neuron array, it was configured with 4k neurons and 80k axon modules. Delay programming and delay adaptation were both used with a pattern length of 51 spikes. For a pattern length of 51 spikes, we tested storing and recalling 1000 and 1200 patterns. Ten test runs were conducted. The system works well for the 1000 pattern case. Figure 12 shows the results for 1000 patterns and the successful recall rate is about 95% on average which is quite close to the result of the fully digital implementation (Wang et al., 2013b). With 1200 patterns the recall no longer works as the effect of cross-talk becomes too severe, indicating that once cross-talk reaches a critical level, it quickly becomes catastrophic. Two reasons caused this performance drop. The first reason is that the mixed-signal system suffers more noise compared to the fully digital implementation, the successful rate of which is 95% for 1200 patterns. The second reason is that the theoretical maximum firing rate of the pre-synaptic spikes that the multiplexed analog neuron array can handle is only 50/128 ≈ 40% of the maximum firing rate that the digital one can handle, as the number of the physical neurons is only 50, whereas the digital implementation has 128 physical neurons.

### Analog axon array and digital neuron array

Unlike the results presented in section Digital axon array and Analog Neuron Array, the testing scenarios for the combination of analog axon array and digital neuron array will focus on the percentage of spikes in a pattern that have been recalled successfully. This is because the capacity of the analog axon array is much smaller than that of the digital axon array, which means that only a few patterns can be stored in this network, so that the percentage of patterns recalled is a much less accurate measure of performance. Furthermore, the dynamics caused by process variation and device mismatch causes variations in the number of spikes that are correctly recalled in each pattern.

For this test, we only had access to one analog axon array with 100 analog axon modules, each with 4 axonal delay paths. The maximum accessible address of the 4/9-bit bus on the analog axon array is 126, which means the maximum size of the digital neuron array that can be used is 126 neurons. As the experimental results in Wang et al. (2013b) show, a neural network consisting of only 126 neurons will be affected seriously by cross-talk. To measure the performance of the analog axon array without the effect of this cross-talk, we used specially generated random patterns with no overlap (correlation) for testing.

Delay programming and delay adaptation were both used with pattern lengths of 20, 25, 33, and 50 spikes. The patterns were trained with a single presentation in the delay programming mode and for 20 presentations in the delay adaptation mode. As there are 400 axons in the analog axon array, for the pattern length of 20, 25, 33, 50 spikes, the maximum number of such patterns that can be stored in the neural network is five, four, three, and two, respectively. For each pattern length, 127 test runs were conducted.

**Figure 13:**
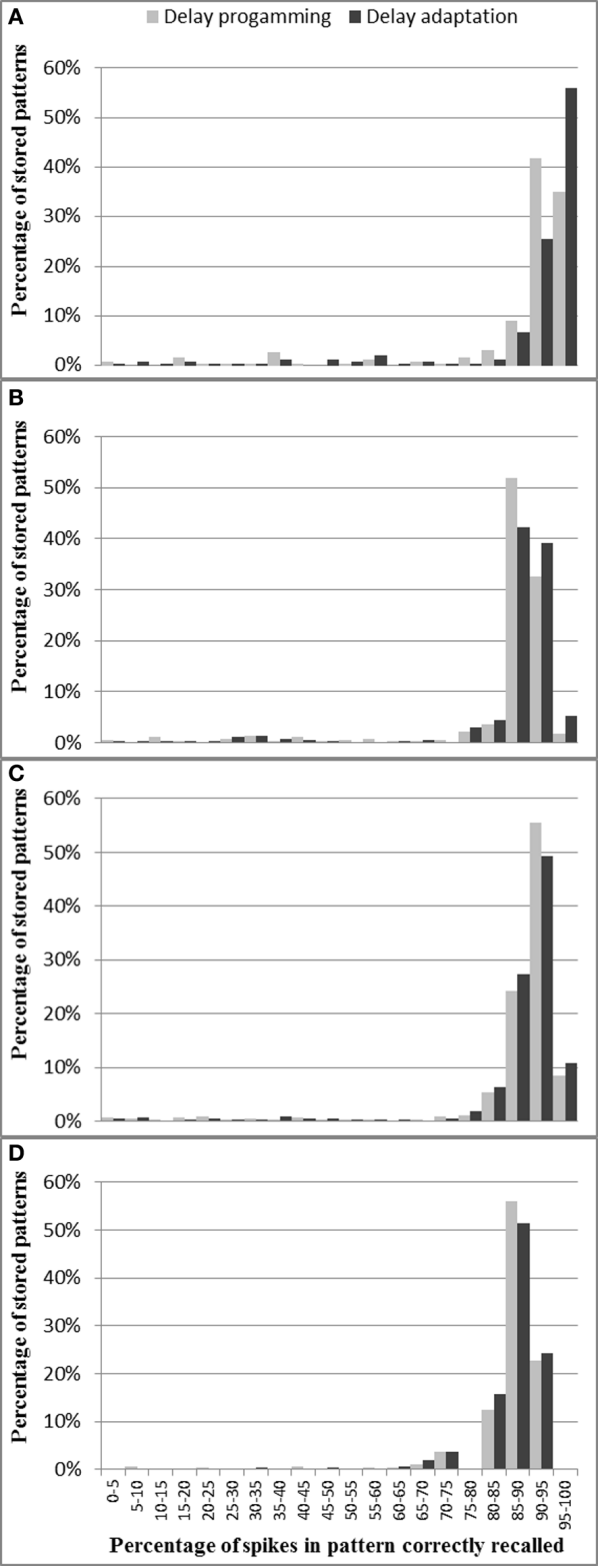
**Percentage of spikes in pattern correctly recalled for different pattern lengths: (A) 50 spikes, (B) 33 spikes, (C) 25 spikes, and (D) 20 spikes**. These results are from the combination of analog axons and digital neurons. For most patterns across all four pattern lengths, more than 85% of spikes are recalled successfully.

Figure 13 shows, for each pattern stored in the neural network, what percentage of spikes were recalled correctly. As discussed in section Analog Axon Array, the delay of the analog axon is programmed in an initial phase followed by a number of iterations of delay adaptation with a fixed delay update step. This is to reduce the errors in delay that result from the initial delay programming step. Figure 13 shows that after 20 iterations of delay adaptation, the percentage of the spikes in the patterns that have been correctly recalled has been slightly increased for the patterns with 50 spikes. For the other pattern lengths, the improvement is negligible. The average percentage of spikes in each pattern correctly recorded across four pattern lengths (over 127 test runs) using delay programming is 86.2% and using delay adaptation is 87%.

Compared to the test results presented in Wang et al. (2013b), which uses the fully digital implementations, the combination of analog axon array and digital neuron array has an 8% drop in performance, which is mainly because the analog axon cannot be as precisely programmed and tuned as the digital axon. As the experimental results of one axon module presented in (Wang et al., 2013a) show, the offset between the actual programmed and the desired value is about 10%, after delay programming. When the ramp generator's voltage is latched by the analog memory (for delay programming), there is always a slight deviation (~10 mV) between the programmed voltage and the desired voltage, as a combined result of charge injection (Liu et al., 2002) and the inaccuracy of the ramp generator itself. The ramp generator will not charge at exactly the same speed each time due to noise in the charging current. The analog axon will therefore propagate each incoming pre-synaptic event with an offset compared to the desired axonal delay. After delay adaptation, this error can be reduced to less than 300 μs throughout the working range of a single axonal delay path (Wang et al., 2013a), but due to process variation and device mismatch, it is impossible to tune all axonal delay paths with such accuracy. This offset, when large enough, will destroy the time-locked relations that are the basis of polychronous spiking neural networks. We will discuss possible solutions for this issue in section Analog vs. Digital Implementations. Another factor in the drop in performance is the fact that the analog axon will sometimes generate spikes due to on-chip parasitic coupling between axons, so that the firing of one axonal delay path can trigger its neighboring paths to fire by accident.

### Analog axon array and analog neuron array

In this section, we will present the experimental results of the combination with an analog axon array and an analog neuron array. For the same reasons as presented in the previous section, the testing scenarios will also focus on the percentage of spikes in a pattern that have been recalled successfully, and the setup for testing is the same as described in the previous section.

**Figure 14:**
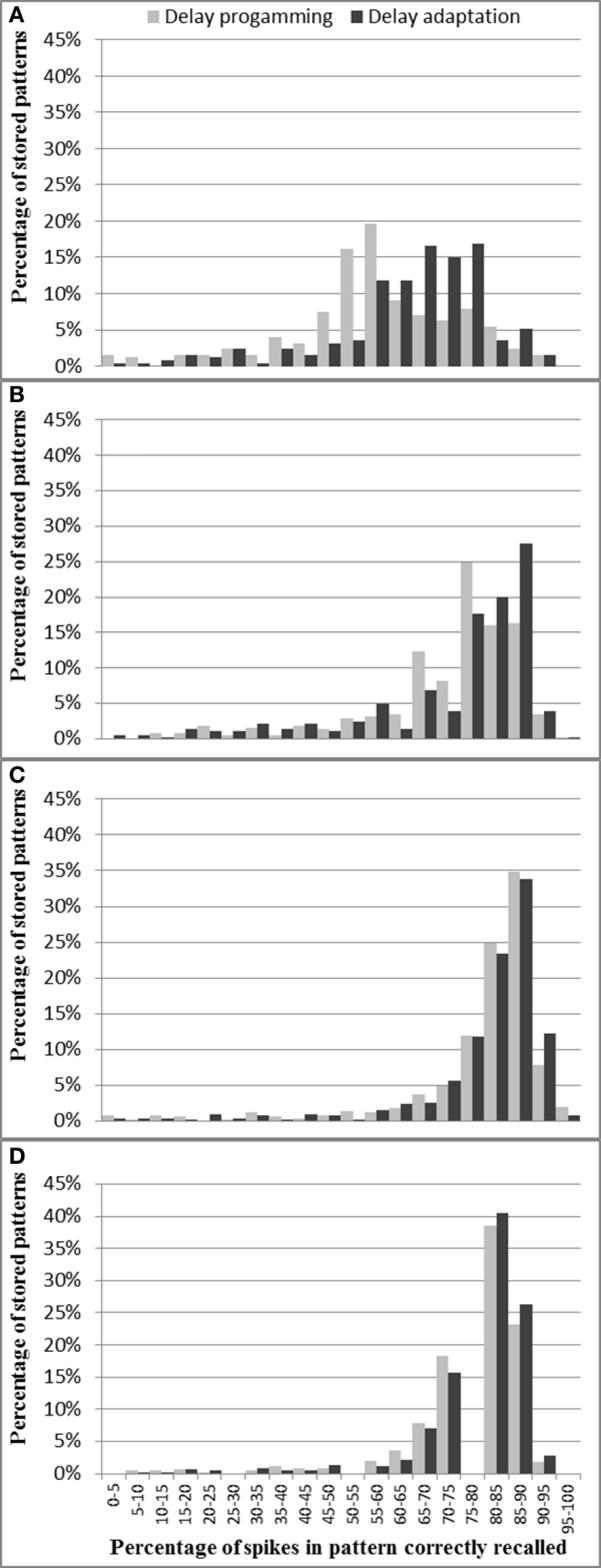
**Percentage of spikes in each pattern correctly recalled for different pattern lengths: (A) 50 spikes, (B) 33 spikes, (C) 25 spikes, and (D) 20 spikes**. These results are from the full analog system.

Figure 14 shows for each pattern stored in the analog axon array how many spikes were recalled correctly. Figure 14 shows that more than 70% of the spikes are correctly recalled for nearly all the patterns across three pattern lengths (20, 25, and 33 spikes) in both delay programming mode and delay adaptation mode. For the longest patterns (50 spikes) the probability of correctly recalling the full pattern is significantly lower, with only 57.4% of the spikes successfully recalled on average, as mismatch and noise are more likely to destroy the time-locked relations, resulting in the final part of the pattern not being recalled. Figure 14 also shows that for these longest patterns, 20 iterations of delay adaptation improve the percentage of the spikes in the patterns that have been correctly recalled to 64.7%. The average percentages of spikes in pattern correctly recorded across four pattern lengths (20, 25, 33, and 50 spikes) using delay programming are 77.2, 78, 72.8, and 57.4%, respectively. After 20 iterations of delay adaptation, these numbers have been improved to 78.1, 78.6, 73.9, 64.7%, respectively. Compared to the results presented in section Analog Axon Array and Digital Neuron array for the analog axon array and digital neuron array, the fully analog combination has an overall Performance drop of about 14%. Compared to the test results presented in section Digital Axon Array and Analog Neuron Array for the digital axon array and analog neuron array, the performance drop increases to about 20%.

These drops are the results of two major factors. The first one is that the analog axon and neuron arrays both generate spurious spikes due to on-chip parasitic coupling. The second factor is that the analog axon fails to perfectly produce the time-locked relations as the digital axon does. Both factors play a larger role the longer the pattern is (in terms of number of spikes). Together, these effects causes the combination of the analog axon and analog neuron array to have the lowest performance of the four combinations.

## Discussion

### Performance comparison

#### Efficiency of the implementation

In Izhikevich (2006), the polychronous network is created with random delays, and STDP is used to prune the connections. Patterns are not stored or programmed into the network, but rather, random patterns emerge. A single connection between neurons could be active in a number of patterns, while other connections will become totally inactive. In our implementation, patterns can be directly programmed into the network and all connections are used when the maximum number of patterns has been programmed into the network. We aimed to avoid inactive connections, since hardware would still be dedicated to these inactive connections, but never used.

A drawback of a polychronous neural network is that a common sequence of four spikes in multiple patterns would initiate all patterns that have this sequence when it occurred. To distinguish between two patterns with identical sub-sequences, it will be necessary to set up the network so that continuous input is needed from the input pattern to keep the pattern going, for example by setting the threshold to 5 simultaneous input spikes (4 from the previous neurons in the pattern and 1 from the input). Such a system would then only follow a pattern if it had been previously learned, and if it corresponded with the input pattern. One of the two potential patterns (with identical starts) would die out once the input signal identified which of the two patterns is being presented.

The probability of overlap between patterns can be reduced by setting a higher threshold at each neuron and connecting it to more of the previous neurons in the pattern. The number of patterns a network can store decreases linearly with the number of neurons each neuron is connected to, so this would come at the cost of a decreased storage capacity.

#### Analog vs. digital implementations

The experimental results show that, on average, the fully digital implementation has the best performance. For comparison, the combination of the digital axon array and the analog neuron array achieves a similar performance when the network is sparse. The combination of the analog axon array and digital neuron array has a considerable performance drop, even when care has been taken to remove all cross-talk from the spatio-temporal patterns. Finally, the combination of the analog axon and neuron array has the worst performance out of the four combinations. The fully digital implementation has the strongest time-locked relation, whereas the fully analog implementation has the weakest, due to the offset between the actual programmed and the desired delay during programming; and the analog implementation is further hampered by noise and spurious spikes. As a result, we may conclude that the most important requirement of a hardware implementation of a polychronous network is to provide a strong time-locked relation.

For the analog axon, as presented in section Analogue Axon Array and Digital Neuron Array, the error is introduced when the ramp generator is writing its output voltage to the analog memory (for delay programming) as a combined result of the charge injection and the inaccuracy of the ramp generator. As the results presented in Wang et al. (2013a) show, the offset will still be about 300 μs even after adaptation. One possible solution is to use analog-to-digital conversions and then store these digital values in digital memories (Horio et al., 1990; Cauwenberghs, 1996). This method has a major advantage in that data can be stored in non-volatile digital memory. The drawback is also quite obvious. It requires at least one analog-to-digital converter (ADC) for storage and usually one digital-to-analog converter (DAC) for read out. This problem will become critical when massive storage is required as each analog cell will either have its own ADC or share one ADC, which will increase the complexity of the circuit. Other factors, such as the accuracy and the bandwidth of the converters, will lead to the requirement for a high precision ADC. The second possible solution is to use floating-gate devices, which employ programmable elements that that could be used to store the analog values in a non-volatile memory (Basu et al., 2010; Brink et al., 2013; Hasler and Marr, 2013). This feature is a promising alternative for the implementation of our polychronous spiking neural network. On the other hand, the time-multiplexed digital axon achieves an excellent balance between hardware cost and performance and therefore is the preferred choice when using FPGAs. As for a custom design, this design choice needs to be carefully investigated because the cost will be highly process dependent.

While it is common cause in neuromorphic engineering that analog circuits provide superior simulation of biological neurons as a result of their continuous and noisy representation of signals, these results show that in this application the analog implementation is consistently poorer in performance and scalability than the digital implementation, which emphasizes that practitioners should recognize that the use of analog circuits comes at a significant cost and should not necessarily be an automatic choice in all applications.

#### Comparison with other solutions

For the analog implementation of the axonal delay, a similar approach was implemented by charging a capacitor using a transistor operating in sub-threshold (Dowrick et al., 2013), so that the duration of the delay can be programmed by adjusting the gate voltage of the charging transistor. However, their implementation is not able to learn delays, as the value of the gate voltage was assigned externally and the authors have not addressed the issues of obtaining and maintaining this voltage. In contrast, our circuit is capable of learning and storing the axonal delay between two spikes. In (Sheik et al., 2012, 2013), the authors show how slow dynamics of analog synapses, combined with the variability of neuromorphic analog circuits, can be used to generate a range of temporal delays. Again, this work is used to generate the desired delay rather than learn the delay.

For the digital implementation of the (axonal) delay, another approach is to use a look-up table for the axonal delay values and use a delay sorter directly before the neurons (Scholze et al., 2011). The delay sorter records the arrival time of a spike and will re-emit the spike when the axonal delay time found in the look-up-table is reached. Our polychronous network generates delay paths *de novo*, so that only connections that actually appear in the training patterns will be created. Each axon module of our polychronous network not only propagates the post-synaptic spike with a programmable axonal delay but also transmits the pre-synaptic spike to the destination neuron (using address remapping by configuring the input and output addresses). An implementation with a look-up table would need the axon module to store the address of the desired axonal delay from the look-up-table, and would need to receive the notification from the look-up-table when that axonal delay is reached. Address-remapping would then have to be carried out by the axon module through the configuration of its input and output addresses. An implementation using look-up tables would therefore be more complex and larger than our proposed implementation.

### Scaling

The performance of the proposed polychronous network (the number of storable patterns) will scale linearly with the number of axons as long as the average number of connections per neuron is kept below 1/4 of the number of neurons in the network to ensure that cross-talk is not much of an issue (Wang et al., 2013b). In other words, the number of neurons needs to be increased proportionally to the number of axons to maintain performance.

The fully digital implementation of the polychronous neural network is a scalable design. The number of time-multiplexed axons implemented by one physical axon will increase linearly with the amount of available on-chip SRAM, as long as the multiplexing rate keeps the time resolution of the system within the biological time scale, which is generally less than 1 ms. The number of physical axons (i.e., the ones that could be activated simultaneously) will increase linearly with the number of available Slice LUTs, which is indeed the bottleneck for large-scale FPGA designs. The total number of virtual axons therefore scales linearly with the quantity of both the available on-chip SRAM and Slice LUTs. The number of physical neurons also scales directly with the number of the available Slice LUTs. Finally, the timing requirement will become quite critical when the utilization becomes high, e.g., 90% of the LUTs on an FPGA, due to the difficulties in routing. A good balance between the number of the physical axons, the multiplexing rate and the number of physical neurons is therefore the key to the implementation of a large-scale polychronous network with a good time resolution and a high utilization of the available hardware recourses on FPGA.

The analog implementation is nowhere near as scalable as the digital implementation, since it can only be scaled up by implementing more physical copies of the neurons and axons. However, the introduction of the multiplexed analog neuron array, making use of the fact that only a few neurons are active at any given time in a polychronous network, allows the number of virtual neurons to be about 80 times larger than the number of physical neurons. In systems that need slow dynamics or memory of past events, i.e., using neurons with longer time constants than we have used here, the multiplex rate would go down and we would need more physical neurons.

### Lessons learned

Some lessons have been learnt from the implementation of this mixed-signal platform and these are discussed below.

Virtualization, i.e., the mapping of a larger address space onto a smaller number of physical components through multiplexing these components, is one of the key ideas for implementing large-scale spiking neural networks, because physical components are costly. Virtualization, when simulating neural networks, is supported by biological observations that only 1% of neurons in our brains are active on average at any moment (Johansson and Lansner, 2007), which means it is not necessary to implement all neurons physically on silicon.

A mixed-signal system appears to be a powerful tool for real-time emulation of large-scale neural networks as it can use analog circuits for computation while keeping the flexibility of using programmable devices such as FPGA. As the on-chip topology of the analog circuits is generally fixed after fabrication, it is better to implement the whole system in an FPGA for prototyping and optimization before fabricating the analog circuits.

For the sake of multiplexing analog building blocks such as neurons and axons in a neuromorphic system, these circuits must be designed as standardized building blocks with a standard protocol for communication (such as AER) with programmable devices. Furthermore, for the maximum utilization of a fixed sized analog chip, it is best to reduce the on-chip routing as much as possible as the routing can be carried out off-chip by FPGAs with more flexibility and extensibility.

Our polychronous network stores spatiotemporal patterns. A certain amount of jitter can be tolerated in the initial spikes when recalling a stored pattern, which is controlled by setting a time window for coincidence detection in the FPGA implementation, and by the neuronal time constant in the analog implementation. If the patterns are to be generated by a neuromorphic sensor, then care needs to be taken that the sensor reliably produces (near) identical spatiotemporal patterns for identical input signals.

## Conclusions

We have presented a mixed-signal implementation of a polychronous spiking neural network composed of both an analog implementation and a digital implementation of the axon array and the neuron array. A multiplexed analog neuron array with 4k analog neurons was achieved by multiplexing 50 physical analog neurons. Compared to conventional time-multiplexing systems that operate serially and have to store and retrieve analog variables, our scheme operates in parallel, and does not require analog storage. A novel interface circuit for synchronizing the spikes from the analog circuits has also been presented. The proposed interface circuit effectively eliminates the problems of timing skew and glitches on the bus and is capable of sampling the asynchronous spikes from the analog circuits correctly. The test results using the four possible configurations of analog or digital components have been compared and discussed. We compared our mixed-signal implementation with our fully digital implementation and addressed the key factor that most influences the performance of the neural network—that of generating accurate time locked relations. The proposed implementation can be linearly scaled up with the quantity of available hardware resources, although the digital implementations are significantly easier to scale than the analog equivalents, owing to the generic FPGA platforms used.

### Conflict of interest statement

The authors declare that the research was conducted in the absence of any commercial or financial relationships that could be construed as a potential conflict of interest.
